# Utility of the Interferon-Gamma Enzyme-Linked Immunosorbent Spot Assay to Predict Risk of Cytomegalovirus Infection in Kidney Transplant Recipients

**DOI:** 10.3389/ti.2023.11527

**Published:** 2024-01-05

**Authors:** Warunyu Namsiripongpun, Surasak Kantachuvesiri, Jackrapong Bruminhent

**Affiliations:** ^1^ Division of Infectious Diseases, Department of Medicine, Faculty of Medicine Ramathibodi Hospital, Mahidol University, Bangkok, Thailand; ^2^ Division of Nephrology, Department of Medicine, Faculty of Medicine Ramathibodi Hospital, Mahidol University, Bangkok, Thailand; ^3^ Ramathibodi Excellence Center for Organ Transplantation, Faculty of Medicine Ramathibodi Hospital, Mahidol University, Bangkok, Thailand

**Keywords:** cytomegalovirus, cell-mediated immunity, immune monitoring, immunocompromised, solid organ transplant

## Abstract

Non‐specific interferon‐gamma (IFN‐γ) enzyme‐linked immunosorbent (ELISpot) responses after solid organ transplant (SOT) and their relationship with cytomegalovirus (CMV) reactivation have hardly been investigated. Adult kidney transplant (KT) recipients underwent measurement of IFN‐γ‐producing T cells using the ELISpot assay before and 1 month after transplantation. Data for CMV infection episodes were collected. Risk factors for post‐transplant CMV infection, based on IFN‐γ responses, were analyzed using a Cox proportional hazards model. A total of 93 KT recipients were enrolled in the study and 84 evaluable participants remained at 1 month post KT. Thirty-three (39%) recipients developed subsequent CMV infection within 6 months post‐transplant. At 1‐month post‐transplant, IFN‐γ‐producing T cells with <250 spot‐forming units (SFUs)/2.5 × 10^5^ peripheral blood mononuclear cells (PBMCs) were significantly associated with CMV infection (HR 3.1, 95% CI 1.4–7.1, *p* = 0.007). On multivariable analysis, posttransplant IFN‐γ‐producing T cells with <250 SFUs/2.5 × 10^5^ PBMCs remained independently associated with CMV infection (HR 3.1, 95% CI 1.2–7.8, *p* = 0.019). Conclusions: KT recipients with low IFN‐γ‐producing T cells measured by the ELISpot assay are more likely to develop CMV infection after transplantation. Therefore, measurement of nonspecific cell-mediated immunity ELISpot responses could potentially stratify recipients at risk of CMV infection (Thai Clinical Trials Registry, TCTR20210216004).

## Graphical Abstract

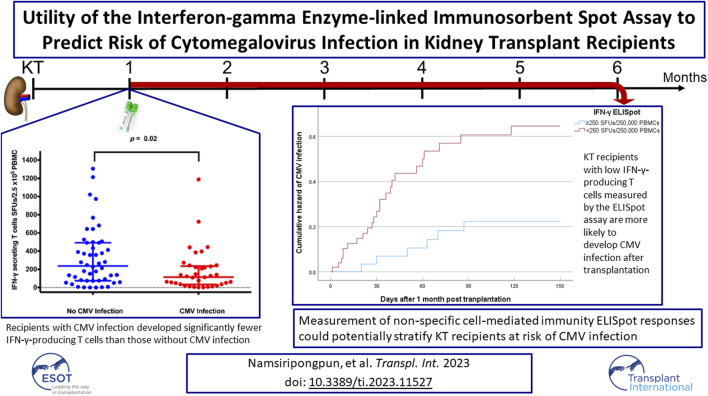

## Introduction

Kidney transplantation (KT) has been widely performed over the past few decades and has improved quality of life and long-term survival among end-stage kidney disease patients requiring renal replacement therapy [1–3]. Immunosuppressants are administered to KT recipients to maintain allograft function and avoid rejection [4]. Although immunosuppressive drugs, especially those that suppress cell-mediated immunity (CMI), provide the advantage of maintaining allograft function, they also place these vulnerable patient populations at increased risk of infection, especially opportunistic infection, after transplantation [5, 6]. As a result, clinicians need to balance the beneficial and deleterious effects of immunosuppressive therapy. Therefore, therapeutic drug monitoring is routinely performed during the course of transplantation to indirectly quantify the net immune status because subtherapeutic and supratherapeutic levels of immunosuppressants are correlated with allograft rejection and viral reactivation, respectively.

There has also been heightened interest in direct measurements of individual immunity. Interferon-gamma (IFN-γ) is an important cytokine with a significant role in antimicrobial and antiviral immunity [7]. Therefore, direct immune status evaluation through measurement of pathogen-specific or non-pathogen-specific IFN-γ-producing T cells has been proposed as a modality to predict specific types of infection in immunocompromised patients. The enzyme-linked immunosorbent spot (ELISpot) assay for IFN-γ measurement has been used for assessment of T cell immunity in response to stimulator cells from donors or third parties in solid organ transplant (SOT) recipients, and has been shown to predict poor long-term renal function in previous studies [8, 9]. However, data regarding non-specific IFN-γ ELISpot production responses to quantify the net state of immunosuppression from an infectious disease perspective are scarce. In the present study, we aimed to determine the utility of the IFN-γ ELISpot assay for measuring cellular immune responses and its correlation with post-transplant cytomegalovirus (CMV) infection in KT recipients.

## Patients and Methods

### Population

A prospective clinical trial of adult KT recipients aged ≥18 years was conducted at Faculty of Medicine Ramathibodi Hospital, Mahidol University, Bangkok, Thailand, between December 2020 and December 2021. The inclusion criteria were adult patients who underwent KT during the study period. The exclusion criteria were surgical postponement regardless of etiology and inadequate peripheral blood mononuclear cells (PBMCs) from venous blood samples. Patients who provided informed consent were monitored clinically for 6 months post-transplant. Study-specific blood samples were collected prior to KT surgery and receiving induction therapy then at approximately 1 month after transplantation to assess for prediction of subsequent CMV infection. Data on demographic characteristics, comorbidities, transplantation types, immunosuppressive therapies, risk factors, and clinical outcomes were collected. Clinical outcomes of interest included CMV DNAemia, CMV syndrome and CMV end-organ CMV disease.

CMV-seropositive KT recipients underwent preemptive CMV monitoring every 2–4 weeks by plasma CMV quantitative real-time polymerase chain reaction (qPCR) assays [CAP/CTM CMV (Roche, Branchburg, NJ, United States) or RealTime CMV (Abbott, Des Plaines, IL, United States)], or when clinically indicated, during the first 3 months. CMV-seromismatched (CMV-seronegative recipient receiving an allograft from CMV-seropositive donor) KT recipients or those who received anti-thymocyte globulin (ATG) for induction therapy or steroid-refractory rejection were provided intravenous ganciclovir or oral valganciclovir for anti-CMV prophylaxis for a period of 3–6 (CMV-seromismatched recipients) months, or were switched to preemptive CMV monitoring for 3 months by plasma CMV qPCR if they were unable to complete the course of therapy. According to our institutional guideline, CMV DNAemia is treated if CMV viral load is greater than 3,000 copies/mL. Both CMV DNAemia and CMV disease patients are treated with intravenous ganciclovir. Preemptive urine screening (i.e., urinalysis and urine culture) is routinely performed on days 3, 7, 10, and 14 after KT then twice weekly until additional 14 days following urinary stent or catheter removal. Trimethoprim/sulfamethoxazole (1 year or longer) for *Pneumocystis jirovecii* prophylaxis, acyclovir (6 months) for herpes simplex virus prophylaxis, and isoniazid (9 months) for latent tuberculous infection therapy were prescribed to all recipients.

The primary objective of the study was to determine the clinical utility of the non-specific IFN-γ ELISpot assay to measure cellular immune responses against phytohemagglutinin (PHA) and its correlation with post-transplant CMV infection in KT recipients. The secondary objectives were to assess risk factors and incidences of CMV infection within 6 months post-transplant.

### IFN-γ ELISpot Assay

Venous blood samples were collected into two 4 mL tubes containing heparin. Sufficient PBMCs were separated by a Ficoll-Paque centrifugation technique and counted using an automated hematology analyzer. The final cell suspension was prepared at a density of 2.5×10^5^ cells/100 µL. The IFN-γ ELISpot assay used in the study is the positive control part of the T-SPOT.TB assay (Oxford Immunotec, London, United Kingdom). The ELISpot assay was initiated by adding 100 µL of suspension and 50 µL of positive control solution containing PHA (Mabtech, Stockholm, Sweden) to commercially available pre-coated 96-well plates (Mabtech). The plates were incubated in a humidified incubator at 37°C with 5% CO_2_ for 18 h. The distinct dark-blue spots produced as a result of antigen stimulation were evaluated and counted by an ImmunoSpot® Analyzer (Cellular Technology Ltd., Cleveland, OH, United States). The completely-developed assay plates were archived for potential re-examination in case of anomalies. The numbers of spot-forming units (SFUs) in paired wells were reported per 2.5×10^5^ PBMCs.

### CMV Infection

CMV Infection was diagnosed by clinical, microbiological, radiological, or pathological evidence. The first author determined the infection episode and a final decision was obtained from the corresponding author. Both are infectious disease specialists. CMV infection was defined as the detection of CMV deoxyribonucleic acid (DNA) in plasma and further classified into asymptomatic CMV DNAemia and CMV disease. The latter was subclassified into CMV syndrome or CMV tissue-invasive diseases according to AST IDCOP and the Transplantation Society International CMV Consensus Group [10, 11]. Data for all CMV infection that occurred within 6 months post-transplant were collected.

### Statistical Analyses

The clinical characteristics were analyzed by descriptive statistics. Categorical and continuous variables were summarized as frequency and percentage, mean and standard deviation (SD), or median and interquartile range (IQR) as appropriate. Comparisons of two categorical outcomes were conducted using the chi-square test or Fisher’s exact test. The Mann–Whitney U test or Student’s t-test were used to analyze the differences between continuous outcomes. Numbers of IFN-γ-producing T cells were presented as dot plots with bars representing the median and IQR, as generated by GraphPad Prism 6.0 (GraphPad Software Inc., San Diego, CA, United States). A receiver operating characteristic (ROC) curve was plotted to determine the IFN-γ ELISpot threshold. Clinical and immunological factors associated with CMV infection were analyzed using the Kaplan–Meier survival estimator and Cox proportional hazards model. Purposeful selection process algorithm was utilized by selecting any variable having a clinically significant univariable test at an arbitrary level of 0.1 to be a candidate for the multivariable analysis. Sensitivity analyses were performed by raising the threshold to 2,000 and 3,000 copies/mL. These cut-off values were selected because of its clinical significance according to our institutional guideline. Values of *p* < 0.05 were considered statistically significant. All statistical analyses were performed using SPSS® Statistics 18 (IBM, Armonk, NY, United States) and STATA 18 (StataCorp, College Station, Texas, United States).

### Participant Consent Statement

The study protocol was approved by the Human Research Ethics Committee of Faculty of Medicine Ramathibodi Hospital, Mahidol University, Bangkok, Thailand (approval number: COA. MURA2020/1983). All patients signed an informed consent form before enrollment in the study. The study was registered in the Thai Clinical Trials Registry (TCTR20210216004).

## Results

### Population

**FIGURE 1:**
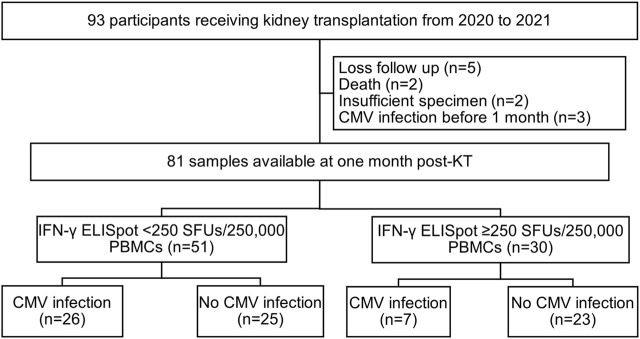
Study flow chart. Abbreviations: KT, kidney transplantation; IFN-γ ELISpot, enzyme-linked immunosorbent spot assay for interferon-gamma; SFUs, spot-forming units; PBMCs, peripheral blood mononuclear cells.

A total of 93 participants were recruited for the study and 81 samples were available for evaluation at 1 month post-transplant (Figure 1). The baseline characteristics of the 93 KT recipients are shown in Table 1. The majority of the recipients received an allograft from a deceased donor (73%) and underwent induction therapy with basiliximab (71%). The maintenance immunosuppression rates were 93% for tacrolimus, 77% for mycophenolate mofetil, and 100% for prednisolone. Almost all participants (98.9%) carried CMV-seropositive status and underwent preemptive CMV DNA load monitoring for 3 months after the transplant. There was only one CMV-seromismatched participant who received ganciclovir prophylaxis for 2 weeks during the hospital stay and then switched to preemptive CMV DNA load monitoring to complete 3 months course. Three episodes of acute rejection occurred on days 6, 23, and 25 after KT.

**TABLE 1:** Baseline characteristics of the 93 kidney transplant recipients.

Characteristics	N (%) or mean ± SD
Female sex	38 (40.9)
Age (years)	44 ± 11
Comorbidities	
Hypertension	76 (81.7)
Diabetes mellitus	11 (11.8)
Hyperparathyroidism	29 (31.2)
HBV infection	4 (4.3)
Unknown	1 (1.1)
Transplant type	
DDKT	68 (73.1)
LRKT	25 (26.9)
BMI (kg/m^2^)	22.6 ± 3.7
CMV serostatus	
D+/R+	88 (94.6)
D−/R+	2 (2.2)
D+/R−	1 (1)
D−/R−	0
Unknown donor CMV status/R+	2 (2.2)
Re-transplantation	7 (7.5)
HLA mismatch	
0	10 (10.8)
1–3	72 (77.4)
4–6	11 (11.8)
PRA (%)	
0–10	71 (76.3)
11–50	10 (10.8)
>50	12 (12.9)
Induction therapy	
Basiliximab	66 (70.9)
Anti-thymocyte globulin	22 (23.7)
None	5 (5.4)
Maintenance therapy	
Tacrolimus	86 (92.5)
Cyclosporine	7 (7.5)
Mycophenolate sodium	21 (22.6)
Mycophenolate mofetil	72 (77.4)
Prednisolone	93 (100)

Abbreviations: SD, standard deviation; HBV, hepatitis B virus; DDKT, deceased-donor kidney transplantation; LRKT, living-related kidney transplantation; BMI, body mass index; CMV, cytomegalovirus; D, donor; R, recipient; +, seropositive; −, seronegative; HLA, human leukocyte antigen; PRA, panel-reactive antibody.

### CMV Infection

Among all 81 evaluable participants at 1 month post KT, 33 (41%) KT recipients developed CMV infection within 6 months post-transplant.

Nearly all CMV infection (30, 91%) were asymptomatic CMV DNAemia. The median (IQR) values of the first and peak CMV DNA load were 784 (223–2,334) and 1,934 (522–7,432) IU/mL. Three CMV diseases comprised one CMV syndrome and two CMV gastrointestinal diseases. The only one CMV-seronegative recipient receiving a CMV-seropositive graft developed CMV syndrome 70 days after transplantation. The patient was admitted and treated with intravenous ganciclovir induction for 1 month leading to clinical resolution and negative CMV viral load before discharge. The median (IQR) duration from transplant to CMV infection was 62 (41–90) days.

### IFN-γ-Producing T Cells and Post-Transplant CMV Infection

The median (IQR) of absolute lymphocytes counts (ALC) before and 1 month after transplantation were 1,104 (861–1,442) and 1,133 (717–1,730) cells/mm^3^, respectively (*p* = 0.42). The median (IQR) numbers of IFN-γ-producing T cells before and 1 month after transplantation were 763 (409–1,067) and 148 (54–389) SFUs/2.5 × 10^5^ PBMCs, respectively (*p* < 0.001). The IFN-γ ELISpot of CMV-seromismatched participant were 395 SFUs/2.5 × 10^5^ PBMCs before KT and 4 SFUs/2.5 × 10^5^ PBMCs 1 month after KT.

**FIGURE 2:**
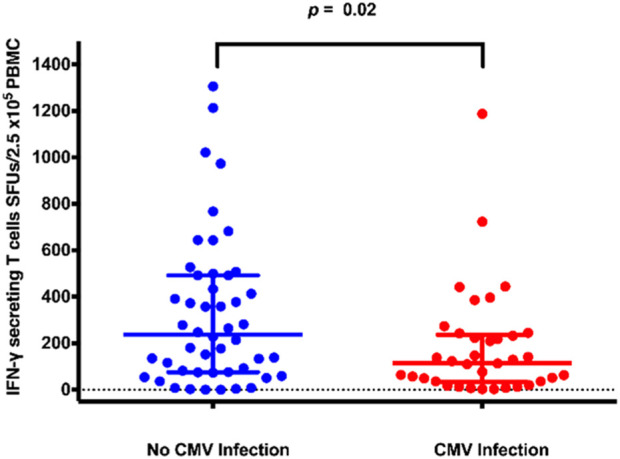
IFN-γ ELISpot distribution plots for kidney transplant recipients with and without CMV infection. Abbreviations: IFN-γ, interferon-gamma; CMV, cytomegalovirus; IFN-γ ELISpot, enzyme-linked immunosorbent spot assay for interferon-gamma; SFUs, spot-forming units; PBMCs, peripheral blood mononuclear cells.

The median (IQR) numbers of IFN-γ-producing T cells at 1-month post-transplant in the KT recipients with CMV infection is presented in Figure 2. Recipients with CMV infection developed significantly fewer IFN-γ-producing T cells than those without CMV infection (115 [33–237] vs. 238 [76–492] SFUs/2.5 × 10^5^ PBMCs, *p* = 0.019).

The ROC curve analysis revealed that the IFN-γ ELISpot assay showed satisfactory test quality to discriminate between CMV infection and no CMV infection with an optimal cutoff value of 250 SFUs/2.5 × 10^5^ PBMCs (AUC 0.65, sensitivity 50%, specificity 80.6%, positive predictive value 66%, negative predictive value 69%), as shown in Table 2. Baseline characteristics of KT recipients classified by IFN-γ ELISpot at 1 month post-transplant were shown in Table 3. Those with IFN-γ ELISpot <250 SFUs/2.5 × 10^5^ PBMCs tend to receive more ATG for induction therapy (27.5%) compared to those with IFN-γ ELISpot ≥250 SFUs/2.5 × 10^5^ (10%).

**TABLE 2:** ROC curve analysis of IFN-γ ELISpot for distinguishing between CMV infection and no CMV infection.

IFN-γ ELISpot cutoff value (SFUs/2.5×10^5^ PBMCs)	Sensitivity (%)	Specificity (%)	Positive predictive value (%)	Negative predictive value (%)	Accuracy (%)
234	50	75	59	67	65
240	50	78	62	68	66
244	50	81	66	69	68
255	48	81	65	68	67

Abbreviations: ROC, receiver operating characteristic; IFN-γ ELISpot, enzyme-linked immunosorbent spot assay for interferon-gamma; CMV, cytomegalovirus; SFUs, spot-forming units; PBMCs, peripheral blood mononuclear cells.

Area under the ROC curve = 0.65 (95% confidence interval 0.53–0.77).

**TABLE 3:** Baseline characteristics of 81 evaluable kidney transplant recipients with IFN-γ ELISpot at 1 month post-transplant <250 or ≥250 SFUs/2.5 × 10^5^ PBMCs.

Characteristics	IFN-γ ELISpot <250 SFUs/2.5 × 10^5^ PBMCs *N* = 51 (%)	IFN-γ ELISpot ≥250 SFUs/2.5 × 10^5^ PBMCs *N* = 30 (%)	*p*-value	Total (*N* = 81)
Female sex	22 (43.1)	12 (40.0)	0.78	34 (42.0)
Age (years)	45 ± 10	41 ± 11	0.07	44 ± 10
Comorbidities				
Hypertension	41 (80.4)	25 (83.3)	0.74	66 (81.5)
Diabetes mellitus	9 (17.6)	1 (3.3)	0.08	10 (12.3)
Hyperparathyroidism	17 (33.3)	8 (26.7)	0.53	27 (30.9)
Transplant type			0.66	
DDKT	38 (74.5)	21 (70)		59 (72.8)
LRKT	13 (25.5)	9 (30)		22 (27.2)
BMI (kg/m^2^)	22.6 ± 3.6	22.1 ± 3.7	0.6	22.4 ± 3.6
CMV serostatus			0.49	
D+/R+	49 (96)	28 (93.4)		77 (95.1)
D−/R+	1 (2)	1 (3.3)		2 (2.5)
D+/R−	1 (2)	0 (0)		1 (1.2)
Unknown donor CMV status/R+	0 (0)	1 (3.3)		1 (1.2)
Re-transplantation	3 (5.9)	2 (6.7)	1.0	5 (6.2)
HLA mismatch			**0.01**	
0	2 (3.9)	7 (23.3)		9 (11.1)
1–3	41 (80.4)	22 (73.4)		63 (77.8)
4–6	8 (15.7)	1 (3.3)		9 (11.1)
PRA (%)			0.43	
0–10	38 (74.5)	26 (86.6)		64 (79)
11–50	6 (11.8)	2 (6.7)		8 (9.9)
>50	7 (13.7)	2 (6.7)		9 (11.1)
Induction therapy			0.12	
Basiliximab	35 (68.6)	24 (80)		59 (72.8)
Anti-thymocyte globulin	14 (27.5)	3 (10)		17 (21.0)
None	2 (3.9)	3 (10)		5 (6.2)
Maintenance therapy				
Tacrolimus	47 (92.2)	30 (100)	0.29	77 (95.1)
Cyclosporin	4 (7.8)	0 (0)	0.29	4 (4.9)
Mycophenolate sodium	13 (25.5)	6 (20)	0.57	19 (23.5)
Mycophenolate mofetil	38 (74.5)	24 (80)	0.57	62 (76.5)
Prednisolone	51 (100)	30 (100)	NA	81 (100)

Abbreviations: IFN-γ ELISpot, enzyme-linked immunosorbent spot assay for interferon-gamma; SFUs, spot-forming units; PBMCs, peripheral blood mononuclear cells; DDKT, deceased-donor kidney transplantation; LRKT, living-related kidney transplantation; BMI, body mass index; CMV, cytomegalovirus; D, donor; R, recipient; +, seropositive; −, seronegative; HLA, human leukocyte antigen; PRA, panel-reactive antibody.

Bold value Indicates the significant p-value <0.05.

### Factors Associated With CMV Reactivation

Cox proportional hazards model analyses were conducted to assess the clinical and immunological factors associated with CMV infection/reactivation within 6 months post-transplant (Table 4). IFN-γ ELISpot <250 SFUs/2.5 × 10^5^ PBMCs was an independent determinant of CMV infection in both univariable and multivariable analyses.

**TABLE 4:** Univariable and multivariable Cox proportional hazards model analyses of clinical and immunological factors associated with CMV reactivation after kidney transplantation.

Factors	Univariable analysis			Multivariable analysis		
	HR	(95% CI)	*p*-value	HR	(95% CI)	*p*-value
Female sex	0.87	(0.44–1.72)	0.697			
Age	1.03	(1.00–1.06)	0.095	1.00	(0.97–1.04)	0.830
BMI	1.06	(0.97–1.16)	0.171			
Hypertension	2.42	(0.74–7.92)	0.143			
Diabetes mellitus	1.38	(0.53–3.56)	0.505			
Hyperparathyroidism	1.12	(0.55–2.29)	0.753			
DDKT	2.09	(0.87–5.05)	0.099	1.65	(0.64–4.25)	0.303
Re-transplantation	0.98	(0.24–4.09)	0.979			
HLA mismatch	0.99	(0.76–1.28)	0.923			
PRA	1.02	(1.01–1.03)	0.001	1.01	(0.99–1.02)	0.538
ATG induction therapy	3.04	(1.53–6.06)	0.002	1.65	(0.42–6.53)	0.472
ALC at 1 month post-transplant ≤500 cells/mm^3^	1.93	(0.84–4.43)	0.119			
IFN-γ ELISpot at 1 month post-transplant <250 SFUs/2.5 × 10^5^ PBMCs	3.30	(1.36–8.03)	0.008	2.83	(1.12–7.13)	0.027

Abbreviations: HR, hazard ratio; CI, confidence interval; BMI, body mass index; DDKT, deceased-donor kidney transplantation; HLA, human leukocyte antigen; PRA, panel-reactive antibody; ATG, anti-thymocyte globulin; ALC, absolute lymphocyte count; IFN-γ ELISpot, enzyme-linked immunosorbent spot assay for interferon-gamma; SFUs, spot-forming units; PBMCs, peripheral blood mononuclear cells.

On univariable analysis, the significant factors associated with CMV infection at 6 months post-transplant were pre-transplant PRA (HR 1.02, *p* = 0.001), ATG induction therapy (HR 3.04, 95% CI 1.53–6.06, *p* = 0.002), and IFN-γ ELISpot <250 SFUs/2.5 × 10^5^ PBMCs (HR 3.30, 95% CI 1.36–8.03, *p* = 0.008). On multivariable analysis, IFN-γ ELISpot <250 SFUs/2.5 × 10^5^ PBMCs was the only significant factor independently associated with CMV reactivation (HR 2.83, 95% CI 1.12–7.13, *p* = 0.027). Harrell’s C value was 0.630 (95% CI 0.573–0.723) with a standard definition of CMV infection. The values increase as we raise the thresholds to 2,000 and 3,000 copies/mL. The C values were 0.694 (95% CI 0.584–0.806) and 0.728 (95% CI 0.623–0.834), respectively.

**FIGURE 3:**
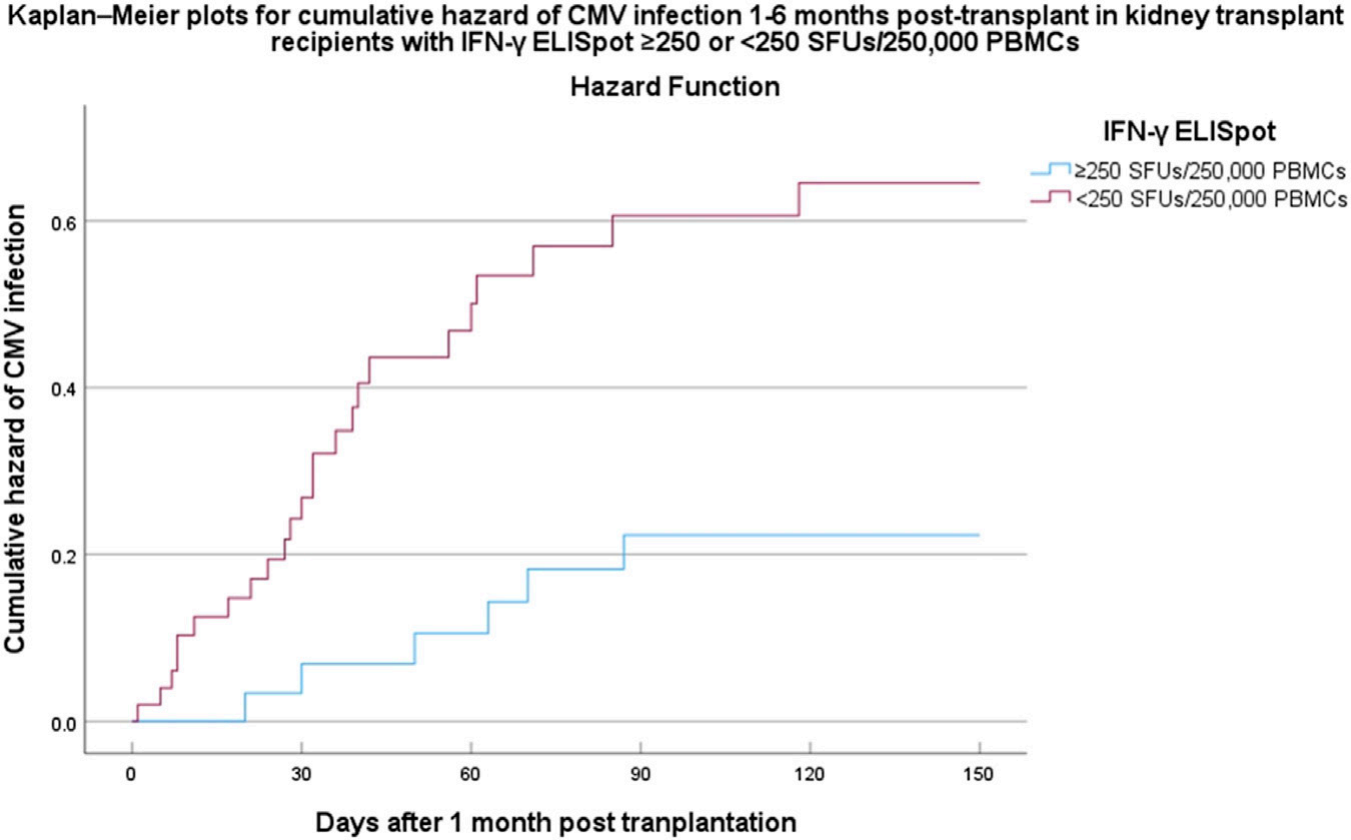
Kaplan–Meier plots for cumulative incidence of CMV infection within 6 months post-transplant in kidney transplant recipients with IFN-γ ELISpot ≥250 or <250 SFUs/2.5 × 10^5^ PBMCs. Abbreviations: CMV, cytomegalovirus; IFN-γ ELISpot, enzyme-linked immunosorbent spot assay for interferon-gamma; SFUs, spot-forming units; PBMCs, peripheral blood mononuclear cells; HR, hazard ratio; CI, confidence interval.

The time to CMV infection stratified by IFN-γ ELISpot (<250 vs. ≥250 SFUs/2.5 × 10^5^ PBMCs) was presented in Figure 3 by a Kaplan-Meier curve (log-rank test < 0.05).

## Discussion

The present study prospectively evaluated non-specific CMI before and after receiving immunosuppressive drugs in KT recipients. IFN-γ-producing T cells after stimulation with PHA were quantified by the ELISpot assay. At a month post-transplant, a significant reduction in IFN-γ-producing T cell responses was observed among KT recipients. Low non-specific CMI, defined as <250 SFUs/2.5 × 10^5^ PBMCs by ELISpot assay, was significantly associated with CMV infection after adjustment for a lymphocyte-depleting agent as induction therapy.

KT recipients are at risk of infection due to the complexities of immunosuppressive medications, instrumentation, and re-transplantation, as represented in our cohort [12]. Among opportunistic pathogens, herpesvirus and polyomavirus are predominant among KT recipients due to the pathogenesis of reactivation under an immunosuppressed state [5]. The significant association with CMV infection could be explained by the containment of this specific pathogen by T cells. The high prevalence of CMV seropositivity in our cohort allowed us to observe this relationship. This association was supported by several previous studies reported in the literature, in which a lack of innate or adaptive immunity was associated with an increased risk of CMV infection in SOT recipients [13–17].

For pathogen-specific immunity, CMV has been widely explored in previous studies. A lack of CMV-specific humoral immunity and CMI before and after transplantation was associated with CMV infection in KT recipients [18, 19]. Specifically, a lack of CMV intermediate early 1–specific CMI, defined as 40 IFN-γ spots/3 × 10^5^ PBMCs at 2 weeks post-transplant, was correlated with CMV infection among KT recipients with basiliximab induction therapy. In the present study, non-specific IFN-γ-producing cells remained independently predictive of CMV infection in a cohort that was mainly composed of recipients with CMV-seropositive status. This finding may be explained by the underlying mechanism for how IFN-γ-producing cells contribute to protection against viral infections, especially the long-term control of viral infections [7, 20]. Immunosuppressants compromise this specific CMI, leading to loss of control and virus reactivation. Although a negative CMV-specific cell-mediated immunity (CMI) measured by QuantiFERON-CMV (QFT-CMV) assay at 1 month after immunosuppressant administration was associated with clinically significant CMV infection in non-transplant immunocompromised (systemic lupus erythematosus) patients with high CMV seroprevalence [21]. The utilization of CMV-specific CMI to predict the risk of infection among CMV-seropositive KT recipients remains to be elucidated and requires further exploration. At least a single time point of the use 1 month post-transplant QFT-CMV assays did not predict CMV DNAemia among KT recipients living in a high seroprevalence setting [22]. Therefore, we proposed that monitoring of overall (non-specific) CMI can better predict KT recipients at risk of CMV infection in the setting where CMV seropositivity is predominant [23].

IFN-γ is an important cytokine synthesized by natural killer cells, CD4 T_H_1 cells, and CD8 cytotoxic lymphocytes of the immune system in response to mitogenic and antigenic stimuli. IFN-γ plays a crucial role in antimicrobial and antiviral immunity [7]. There are several tools to measure the state of immunity in immunocompromised individuals. Virus-specific CMI can be measured by enzyme-linked immunosorbent assay (ELISA), ELISpot assay, or intracellular cytokine staining. Indeed, a lack of CMV-specific IFN-γ -producing T cell responses measured by ELISA, ELISpot, or intracellular cytokine assay was shown to be associated with CMV infection in SOT recipients. We demonstrated that IFN-γ ELISpot response to PHA in KT recipients at 1-month post-transplant was an independent biomarker predictive of CMV reactivation. The IFN-γ ELISpot assay is the positive control part of a commercially available and standardized TB-specific ELISpot assay, and can be routinely performed in a clinical laboratory. IFN-γ was shown to be predictive of acute allograft rejection in a previous study [24]. However, another study found that donor-specific IFN-γ ELISpot was not predictive of allograft loss [25]. The ELISpot assay has an advantage over other assays by measuring extracellular IFN-γ, which is believed to be more functional than measurement of intracellular components. Furthermore, a washing step that is unique to the ELISpot assay procedure may remove pre-existing IFN-γ and other potential substances that could interfere with the results. International guidelines have encouraged the use of these tools to guide clinicians when treating and offering prevention strategies to SOT recipients [10, 11].

Several studies have investigated the role of non-pathogen-specific CMI in predicting the occurrence of CMV infection after transplantation. Immuknow assay, a commercially available assay, which provides an assessment of global cell-mediated immune responses revealed that those with impaired CD4 T cell responses were likely to develop significantly more CMV disease [16]. QuantiFERON monitor assay revealed that IFN-γ in solid organ (including kidney) transplant recipients at 1-month post-transplant was significantly lower in those with CMV disease [26]. Those findings were similar with our study which utilized different global immunity monitoring technique.

On the other way, a simple and practical way to indirectly measure non-specific CMI could be obtained from absolute lymphocyte count (ALC). Lymphopenia with an absolute lymphocyte count of <610 cells/mm^3^ was correlated with an elevated risk of CMV reactivation in SOT recipients [27]. Severe lymphopenia (defined as ALC <500 cells/mm^3^) during pretransplant [15] and early post-transplant periods [17] was an independent risk factor for CMV disease and early CMV infection, respectively. However, we did not observe an increased risk of post-transplant CMV reactivation in KT recipients with an ALC of ≤500 cells/mm^3^. We believe being able to assess CMI function may possibly be a better option to stratify CMV risk in SOT population with CMV seropositivity.

The present study has several limitations. The small sample size and the relatively high proportion of dropouts at 1 month post-transplant were inadvertently aggravated by the COVID-19 pandemic. Only one case of CMV-seronegative recipient receiving a CMV-seropositive graft was recruited in our study. Thus, the correlation between non-specific IFN-γ ELISpot and CMV infection cannot be extrapolated to this transplant subpopulation. The statistically significant differences may not be translated into clinical practice because a quarter of participants with high non-specific CMI still developed CMV infection in our study. Furthermore, many recipients with CMV viral load above institutional threshold were not given antiviral therapy. Decreased immunosuppressive therapy led to resolution of CMV DNAemia in these patients. As a result, non-specific IFN-γ-producing cells should be further assessed in a larger cohort with a longer follow-up duration. The test could also have limited clinical utility because it is technically complicated and not available in a resource-limited diagnostic laboratory. However, we have demonstrated the potential role of overall immune monitoring in predicting CMV infection by the ELISpot assay in KT recipients with profound immunosuppression.

In conclusion, an intact overall net state of CMI in KT recipients early after transplantation is a protective factor against post-transplant CMV infection within the first few months. KT recipients with a low IFN-γ response are more likely to develop CMV infection. Therefore, measurement of non-specific CMI responses using the ELISpot assay could potentially stratify KT recipients at risk of CMV reactivation. Clinicians should be able to design prevention strategies, either by preemptive approaches or prophylaxis, based on the actual immune status in individual recipients.

## Data Availability

The raw data supporting the conclusion of this article will be made available by the authors, without undue reservation.
